# Porous silicon carbide coated with tantalum as potential material for bone implants

**DOI:** 10.1093/rb/rbaa021

**Published:** 2020-06-18

**Authors:** Zhijie Ma, Jingyu Li, Fang Cao, Jiahui Yang, Rong Liu, Dewei Zhao

**Affiliations:** r1 Faculty of Electronic Information and Electrical Engineering, School of Biomedical Engineering, Dalian University of Technology, No. 2 Linggong Road, Ganjingzi District, Dalian, Liaoning 116024, China; r2 Orthopaedic Department Affiliated ZhongShan Hospital of Dalian University, No. 6 Jiefang Street, Zhongshan District, Dalian, Liaoning 116001, China

**Keywords:** porous SiC scaffold, tantalum coating, chemical vapour deposition, bone implant material

## Abstract

Porous silicon carbide (SiC) has a specific biomorphous microstructure similar to the trabecular microstructure of human bone. Compared with that of bioactive ceramics, such as calcium phosphate, SiC does not induce spontaneous interface bonding to living bone. In this study, bioactive tantalum (Ta) metal deposited on porous SiC scaffolds by chemical vapour deposition was investigated to accelerate osseointegration and improve the bonding to bones. Scanning electron microscopy indicated that the Ta coating evenly covered the entire scaffold structure. Energy-dispersive spectroscopy and X-ray diffraction analysis showed that the coating consisted of Ta phases. The bonding strength between the Ta coating and the SiC substrate is 88.4 MPa. The yield strength of porous SiC with a Ta coating (pTa) was 45.8 ± 2.9 MPa, the compressive strength was 61.4 ± 3.2 MPa and the elastic modulus was ∼4.8 GPa. When MG-63 human osteoblasts were co-cultured with pTa, osteoblasts showed good adhesion and spreading on the surface of the pTa and its porous structure, which showed that it has excellent bioactivity and cyto-compatibility. To further study the osseointegration properties of pTa. PTa and porous titanium (pTi) were implanted into the femoral neck of goats for 12 weeks, respectively. The Van-Gieson staining of histological sections results that the pTa group had better osseointegration than the pTi group. These results indicate that coating bioactive Ta metal on porous SiC scaffolds could be a potential material for bone substitutes.

## Introduction

Metal materials have excellent mechanical properties and fatigue resistance, which are especially suitable for bone replacement implants in weight-bearing parts in the human body. Therefore, various metal materials, including cobalt-based alloys, titanium alloys and stainless steel, have been widely used as biomedical materials, and have achieved good therapeutic results. Nonetheless, metal materials have markedly increased Young’s modulus, compared with cortical bone. This may lead to the phenomenon of stress shielding, in which extended bone contact lowers the bone physical load, resulting in embrittlement of bone and even causes secondary fractures [1–3]. In addition, a complex human body environment may give rise to material corrosion and toxic ions release, reducing the biocompatibility of metal materials [4, 5]. For reducing the modulus of elasticity, enhancing the compatibility between bone tissue and implant materials, and accelerating the osseointegration, materials scientists have proposed a method of introducing pores into the material. Compared with dense materials, the density, strength and modulus of elasticity for the porous structure materials can be adjusted to match those of bone tissue mechanical properties by adjusting the structure of the pores. Simultaneously, the porous structure has a unique 3D connected space, which can transfer body fluids and nutrients in porous implant materials, promote tissue regeneration and reconstruction, and form a biological fixation between the implant materials and bone, thereby finally forming as a whole. 

Porous metal materials have become one of the hotspots in biomaterials research. Among them, porous tantalum (Ta) metal has been considered to be an ideal orthopaedic implant material. This new type of biomaterial can be produced by depositing a Ta coating on a porous carbon substrate [6]. This was originally developed by Implex Corp., and named Hedroced. After this was acquired by Zimmer Inc. in 2003, the product was renamed Trabecular Metal. Since its introduction, the application range of pTa has continuously expanded. It was revealed through clinical observation that pTa bone-implanted devices exhibit excellent biocompatibility, and the pTa and bone tissue binds well. Furthermore, the combination cannot easily loosen and exhibits excellent long-term stability [7–9]. However, some publications mention issues, such as the brittle deformation behaviour of pTa [10–12], which limits the separate application of pTa in human weight-bearing parts [13].

In recent years, porous silicon carbide (SiC) has received increasing attention due to its outstanding physicochemical performances, which including corrosion and oxidation resistance, together with strength [14]. The Institute of Metal Research, Chinese Academy of Sciences has developed the porous SiC materials with the use of liquid infiltration–reaction in combination with polymer pyrolysis, and these materials have exhibited high controllability and good mechanical properties. Wu *et al*. reported that the corresponding compressive strength of this porous SiC foam is ∼13–60 MPa, while the flexural strength is ∼8–30 MPa [15]. The high compressive strength helps to maintain the early strength of the bone and meets the requirements for early mechanical strength. In addition, arbitrarily complex components can be fabricated from porous SiC ceramics, and the 3D connected porosity and pore size can be modulated based on human bone strength requirement at different positions. Thus, these results indicate that it is feasible to use the porous SiC to repair complicated and weight-bearing bone defects.

Porous SiC ceramics are superior in various aspects. However, these lack bioactive, and cannot induce new bone formation and osseointegration. In order to compensate for these shortcomings, some scholars have applied bioactive hydroxyapatite and bioactive glass coatings onto porous SiC materials [16, 17]. However, hydroxyapatite and bioactive glass are brittle materials. In clinical practice, the coating may peel off from the weight-bearing substrate material, and which limits its further use [18, 19]. As a result, novel coatings that have excellent osteoinductivity, firm bonding with substrates and long-term chemical stability are still needed for porous SiC scaffolds.

In the present study, the combination of the 3D pore structure similar to the human cancellous bone and the excellent mechanical performances of biomorphic SiC scaffolds, which are used as the base material for the implants, and the osteoconducting properties of bioactive Ta coatings provide new possibilities for the development of alternative bone grafts. The chemical vapour deposition (CVD) system was used to deposit the Ta coating on the porous SiC scaffold. In addition, X-ray diffraction (XRD) and scanning electron microscopy (SEM) were carried out to characterize the microstructure and composition of the Ta coating. In addition, a multi-functional micro-friction wear tester was used to test Ta coating for its bond strength. The mechanical properties were tested using a mechanical testing machine. Human osteoblast cells (MG-63) were used to evaluate the SiC with the Ta coating for its biocompatibility *in vitro*. The Ta-coated porous SiC scaffolds were investigated for its osseointegration property using the goat implantation model (pTa).

## Materials and methods

### Fabrication for ta coating on porous SiC scaffolds

The porous SiC scaffolds were obtained from the Institute of Metal Research, Chinese Academy of Sciences. The bioactive Ta metal was coated onto the porous SiC scaffold using the CVD system. The raw materials for the reaction were high-purity Ta metal (99.99%), high-purity chlorine gas (99.999%) and hydrogen (99.999%) as the reactive gases, and high-purity argon gas (99.998%) as the protective gas. The porous SiC scaffold was initially ultrasonically rinsed to ensure the same surface state. Then, the porous SiC scaffold and the Ta raw material were placed at both ends of the reaction chamber, and a vacuum below 100 mTorr was achieved. The chlorine gas was initially transported to the front end of the chlorination chamber that was heated to 1050°C, and the chlorine gas reacted with the Ta raw material to form Ta pentachloride. Then, the vaporized Ta pentachloride reacted with the hydrogen to form a Ta film that was deposited on the porous SiC scaffold. The deposition was conducted for 28 h. The exhaust gas generated by the reaction reacted with sodium hydroxide in the filtration system to form hydrogen chloride. This was subsequently discharged through a mechanical pump and did not result in environmental pollution. The chemical reaction method was as follows:

(1) $$\text{Ta}+5/{2\;\text{Cl}}_{2}={\text{TaCl}}_{5}$$

(2) $${\text{TaCl}}_{5}+{5H}_{2}=\text{Ta}+5\text{HCl}$$

(3) $$\text{NaOH}+\text{HCl}=\text{NaCl}+H_{2}O$$

After the pTa metal was successfully prepared, all samples were ultrasonically cleaned.

### PTa surface analysis

An energy-dispersive spectrometer (EDS; Oxford INCA energy 300) was used to measure Ta coating for its chemical composition. SEM (HITACHI S-3500, Japan) was used to characterize the surface morphology and microstructure of porous SiC and pTa. Furthermore, XRD (Rigaku D/Max 2500PC, Tokyo, Japan) was applied to characterize the Ta coating structure under CuKα radiation. The MDI Jade 5.0 software (Materials Data Inc., CA, USA) was used to collect XRD patterns.

### Ta coating adhesion

The Ta coating adhesion was tested using a multi-functional micro-friction wear tester (UMT-2M). An YG6 cemented carbide indenter composed of 94% WC and 6% CO with a hardness of HRA90.5 was pressed vertically into the coating at a constant speed and moved horizontally to scratch the surface of the coating. The uniform continuous load was 0.2–20N, the horizontal speed of the indenter was 0.1 mm/s, and the distance was 10 mm. Each sample is divided into three lines, the first line and the second line are 2 mm apart, the second line and the third line are 1 mm apart. The sound signal was collected, and when the signal was abrupt, the film was considered to be broken, and the corresponding load was the critical load. The coating adhesion force was then evaluated by the critical load.

### Mechanical testing

The mechanical test was performed according to the ISO 13314 standard [20]. The compressive strength, yield strength and elastic modulus were measured using a universal testing machine (Hung Ta HT-2402, China). The compression test was performed on pTa samples under 0.2 mm/min until the samples was crushed. The force and deformation data were recorded to generate the stress–strain curves.

### *In vitro* biocompatibility

The MG-63 human osteoblasts were purchased from Beijing Union Cell Bank. After the MG-63 cells were resuscitated, the culture solution was added, and the cells were allowed to stand under 5% CO_2_ atmosphere at a temperature of 37°C. After changing the solution once at an interval of 2–3 days, the inverted microscope was used to observe cell growth when reaching 80% confluence. Then, the cells were digest with 0.25% trypsin solution for 3 min and centrifuged. The sterilized pTa material was cultured in MEM medium (HyClone, USA) supplemented with 10% foetal bovine serum (Corning, USA) for 24 h. Then, this was taken out of the wells of a 24-well culture plate, and 25 μl of MG-63 cell suspension (concentration: 5 × 10^4^ cells/ml) was inoculated to pTa, and cultured under 5% CO_2_ and 37°C conditions. Subsequently, the co-culture of MG-63 cells with pTa materials was carried out for 3 and 7 days. Next, the cell scaffold complex was taken out, followed by phosphate-buffered saline (PBS) washing, 2.5% glutaraldehyde fixation, gradient ethanol dehydration, drying at a critical point and coating with a sputtered Au coating. SEM (HITACHI S-3500, Japan) was used to observe the samples.

### Surgical procedure

Six healthy goats (weighing 25–30 kg, 1-year old, both genders) were obtained from Dalian Medical University. The Animal Care and Experiment Committee at the Affiliated Zhongshan Hospital of Dalian University approved the present animal experimental protocols. These were randomly divided into two groups of pTa and porous titanium (pTi) (one for each three). Ketamine and Sumianxin were mixed at a 2:1 ratio, and 0.5 ml/kg was administered intramuscularly for anaesthesia. Holes were drilled in the femoral greater trochanter on the left side of the goat, and the pTa rods and pTi rods were implanted (with the length and diameter of 30 and 5 mm, respectively). After disinfection, both soft tissue and skin were sutured in order. Penicillin sodium (800 000 U/day) was injected 30 min before surgery and 3 days after surgery, in order to prevent infection. At 12 weeks after implantation, these goats were euthanized by over-injection of pentobarbital.

### Histological analysis

After clearing the soft tissue around the femoral neck of the goat, samples were subjected to 10 days of fixation with 4% paraformaldehyde contained within the PBS, 3 days of gradient dehydration with ethanol, and embedding within the methyl-methacrylate. The Saw Microtome Exakt E300 (Exakt, Germany) was used to cut the embedded samples to 100-μm thick sections, followed by grinding to 50–60 μm in thickness. Finally, Van Gieson staining was used for tissue morphology analysis. The bone formation area (NB) was quantitatively evaluated using Imagetool V3.0 software (UTHSCSA, USA).

### Statistical analysis

The results were expressed in mean±standard deviation (SD) and the differences between groups were compared using one-way analysis of variance (ANOVA) or independent sample *t*-test. *P* < 0.05 was considered statistically significant.

## Results and discussion

### Morphology characteristics of the SiC scaffold and Ta coating

**Figure 1:**
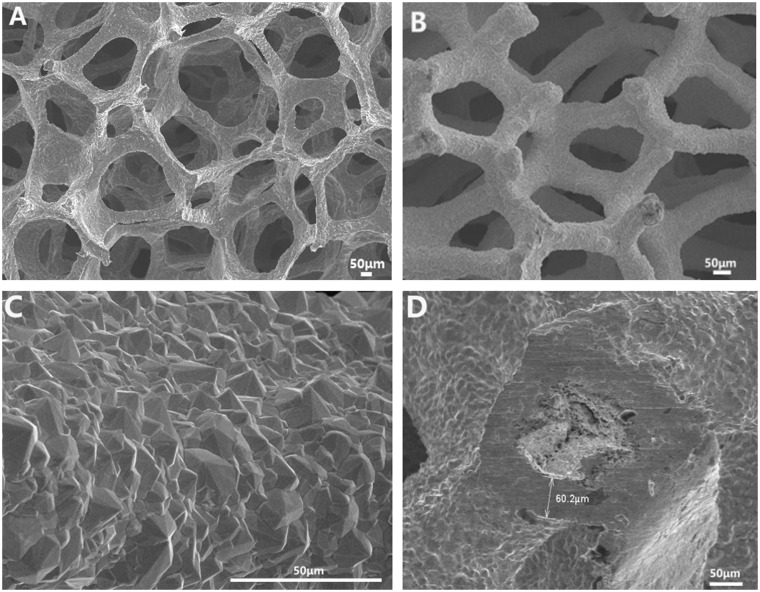
SEM observation of the structure and surface morphology of the pTa and porous SiC scaffold: (**A**) porous silicon carbide scaffold, where the pore structure is similar to that of a human trabecular structure; (**B**) pTa scaffold with fully connected 3D pore structure, where the ta metal was uniformly coated on the SiC scaffold; (**C**) crystal structure and size; (**D**) Ta coating thickness. Scale bar = 50 μm.

Figure 1A shows a porous SiC scaffold, which is similar to the porous structure of a human trabecular bone, with a pore diameter of 550 ± 37 μm. Figure 1B shows that the Ta coating is uniformly deposited on the porous SiC scaffold, the porous scaffold is not exposed, and the pore diameter is ∼370 ± 42 μm. The Ta coating is magnified to 1500 times (Fig. 1C), and it can be seen that the Ta coating is formed by the aggregation of Ta grains. The grain structure is pyramidal, the grain size is uniform and the grain diameter is ∼5–15 μm. The pTa was cut transversely, and the thickness of the Ta coating was measured to be ∼60 μm (Fig. 1D). Compared with non-porous materials, studies [21–24] have shown that porous materials, especially porous materials with a pore size of ∼300–600 µm, are more conducive to cell adhesion, proliferation and differentiation. Without the need for osteoinductive biomolecules, these specific porous structures can induce new bone formation and accelerate osseointegration. The prepared pTa scaffold comprises a dodecahedral porous structure, similar in structure to a human cancellous bone. The pore diameter is maintained at 370 ± 42 μm and the porosity up to 80.2%, which provides favourable growth space for cell and tissue adhesion and growth in addition to growth of new bone tissue. The pyramidal crystal particles lead to a rough pTa surface and are a natural product of CVD; the pTa coefficient of friction is 40–80% higher than that of other surface treatment materials [6]. The high coefficient of friction promotes the close adhesion of cells and tissues to the implant, facilitates the adsorption of macromolecular substances on cells and bone tissue, and enhances the strength of the connection between the scaffold and the bone tissue [13]. It is the first step in the formation of a good coating interface.

### Characterization of the deposited coatings

**Figure 2:**
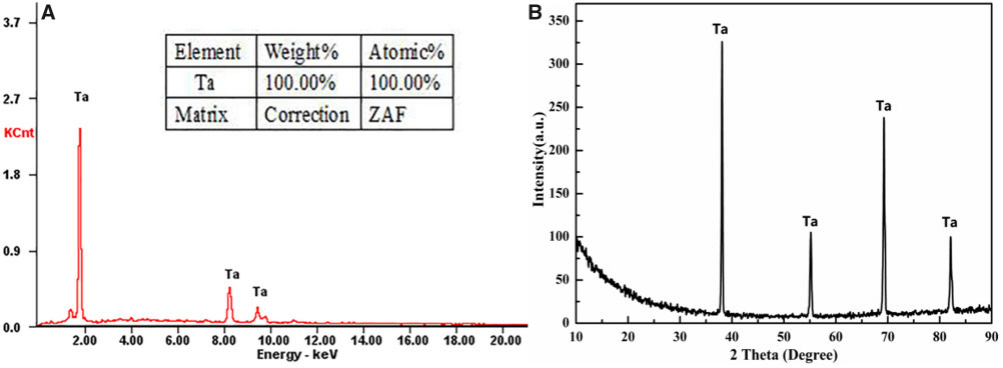
(**A**) EDS survey spectra and (**B**) XRD patterns of the SiC substrate and ta coating.

The crystallographic structures of the Ta coatings deposited on the porous SiC scaffolds were examined using EDS and XRD. The EDS revealed that the major element in the coating was Ta (Fig. 2A). The XRD patterns of the CVD coatings on the scaffolds were indexed as Ta phases, according to the relevant JCPDS card. Thus, the Ta coating was successfully fabricated on the porous SiC substrate (Fig. 2B). The EDS and XRD results prove that the purity of the Ta coating is 100%, which fully demonstrates the advantages of CVD. High-purity pTa metal can maintain long-term chemical stability after being implanted into the body. Furthermore, toxic ions are not released, and this does not have to be removed.

### Ta-coating adhesion

**Figure 3:**
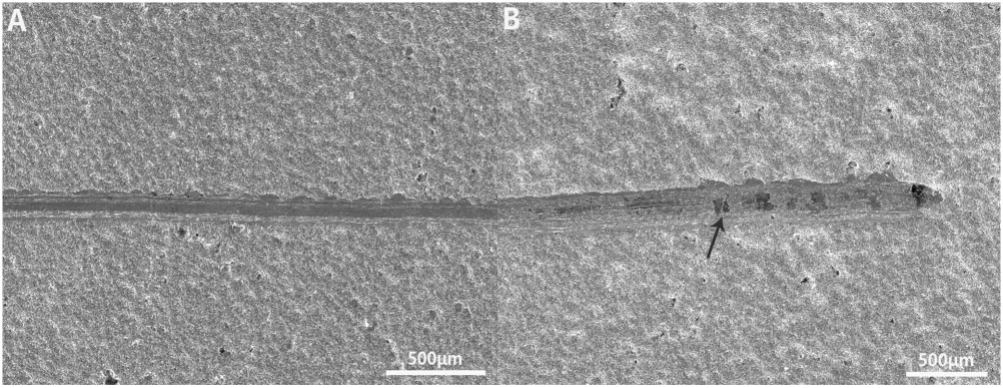
(**A**) Morphology of scratches on the surface of the SiC plate; (**B**) the coating begins to crack (at the white arrow) at the end of the scratch. Scale bar = 50 μm.

The coating adhesion is an important indicator that should be addressed before clinical application. The scratch method is one of the most commonly used methods for measuring the bonding strength of single-layer films. The test results show that the average critical load value of the Ta coating from the silicon carbide (SiC) planar substrate is 20N; that is, the bond strength between the Ta coating and the SiC substrate is 88.4 MPa (Fig. 3). Currently, the ISO standard for hydroxyapatite coatings is that the bond strength must be >15 MPa [25]. The Ta coating adhesion is much higher than the bonding strength requirement for the clinical application of hydroxyapatite coatings and the titanium-based metal bonding strength of 15–25 MPa [26]. The Ta coating prepared by the CVD method has good bonding strength with the silicon carbide substrate, which is mainly due to the high density and hardness of the Ta coating crystal structure. An excellent coating adhesion can effectively prevent the implant coating from delaminating in the body, reduce the probability of chronic loosening of the implant and infection; thus, the adhesion is an important part of forming a good interface.

### Mechanical properties tests

**Figure 4:**
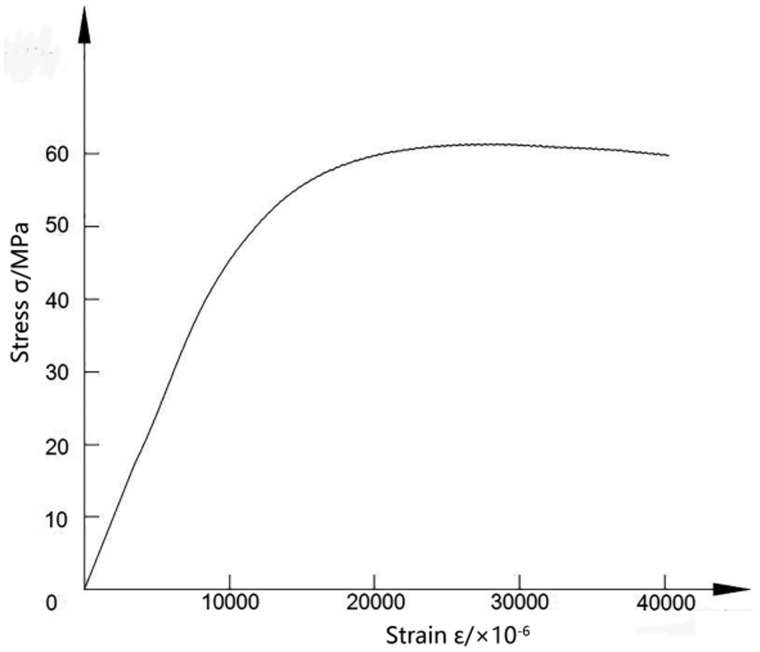
Compressive stress–strain curve of the pTa.

The stress–strain curve from the compressive mechanical properties test of the pTa metal is shown in Fig. 4. The yield strength of the pTa is 45.8 ± 2.9 MPa, the compressive strength is 61.4 ± 3.2 MPa and the elastic modulus is ∼4.8 GPa. Compared with those for pTa metal products from Zimmer Inc., the compressive strength and yield strength are basically the same, but the elastic modulus is higher than the value of 3.1 GPa for the Zimmer Inc. material and is closer to the elastic modulus for human cortical bone (10–30 GPa) [27]. The pTa metal prepared by using porous SiC as a scaffold has excellent compressive strength and yield strength. The elastic modulus is between those for cortical bone and cancellous bone. While satisfying the good mechanical properties of the implant material, it can effectively avoid the adverse effects caused by stress shielding and has the potential to be the ideal bone implant material. At the same time, according to the needs of different weight-bearing parts of human bones, the deposition rate can be adjusted to adjust the thickness of the Ta metal coating, and the mechanical strength requirements of different load-bearing parts of the human body can be met in a cost-effective way.

### Biocompatibility evaluation

**Figure 5:**
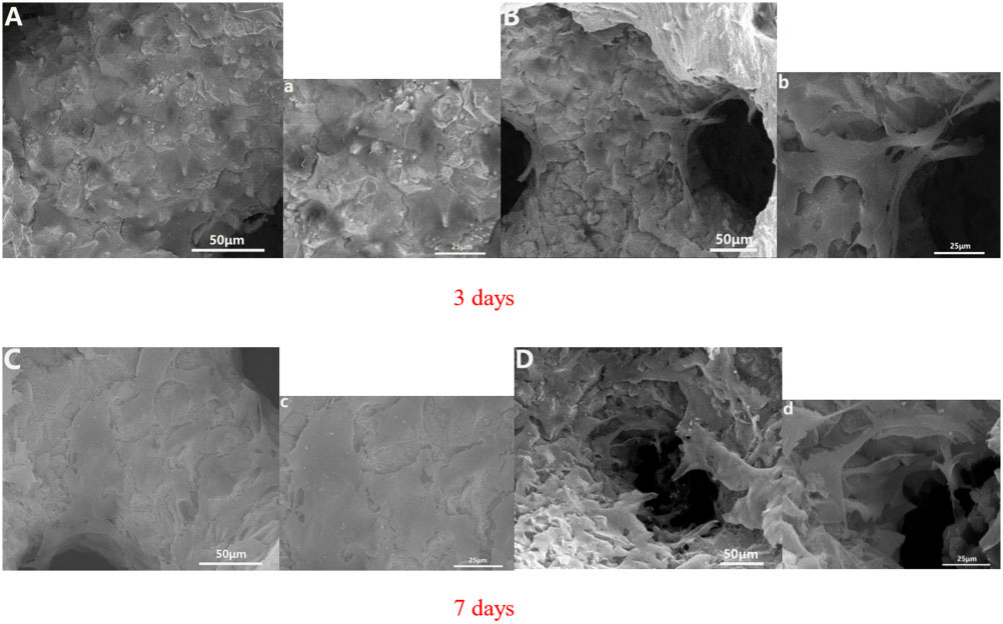
SEM morphology of MG-63 human osteoblast cells on pTa that were co-cultured for 3 and 7 days *in vitro*: (**A**, **C**) the pTa surface cell growth images and (**B**, **D**) cell growth images in pTa pores. The large image was magnified ×500 with a scale bar = 50μm; the small image was magnified ×1000 with a scale bar = 25 μm.

MG-63 human osteoblasts were co-cultured with Ta-coated porous SiC scaffolds. The adhesion and proliferation of cells on the pTa were observed by SEM. After 3 days of co-cultivation, the morphology of the MG-63 human osteoblasts that attached onto the pTa was determined and is shown (Fig. 5). It can be clearly seen that a large number of cells adhere to the surface of the porous SiC scaffold that is coated with Ta (Fig. 5A) and the porous structure (Fig. 5B), and the cells are fully spread on the porous Ta surface and in the pores. Some of the cells protrude from the pseudopod and are connected to each other. After 7 days of co-cultivation, the surface cells of the pTa become cross-linked to each other and the cell protrusions fuse to form a sheet covering the surface of the pTa (Fig. 5C). In the porous structure, the cells protrude from the pseudopods, cross-link to each other across the pores, secrete in the matrix, and coat the microparticle structure, gradually spreading in the pores (Fig. 5D). This shows that the 3D structure and rough surface morphology of the pTa material on the porous SiC as a scaffold facilitate cell adhesion and proliferation. In addition, the surface adhesion and spreading of the osteoblasts is a crucial indicator of implant bioactivity and long-term cellular behaviours (e.g. migration and differentiation). Combined with the activity of the Ta, it was proven that a SiC porous scaffold with a Ta coating could satisfy the requirements for orthopaedic application because this structure could promote bone healing and reconstruction.

### Histological analysis

**Figure 6:**
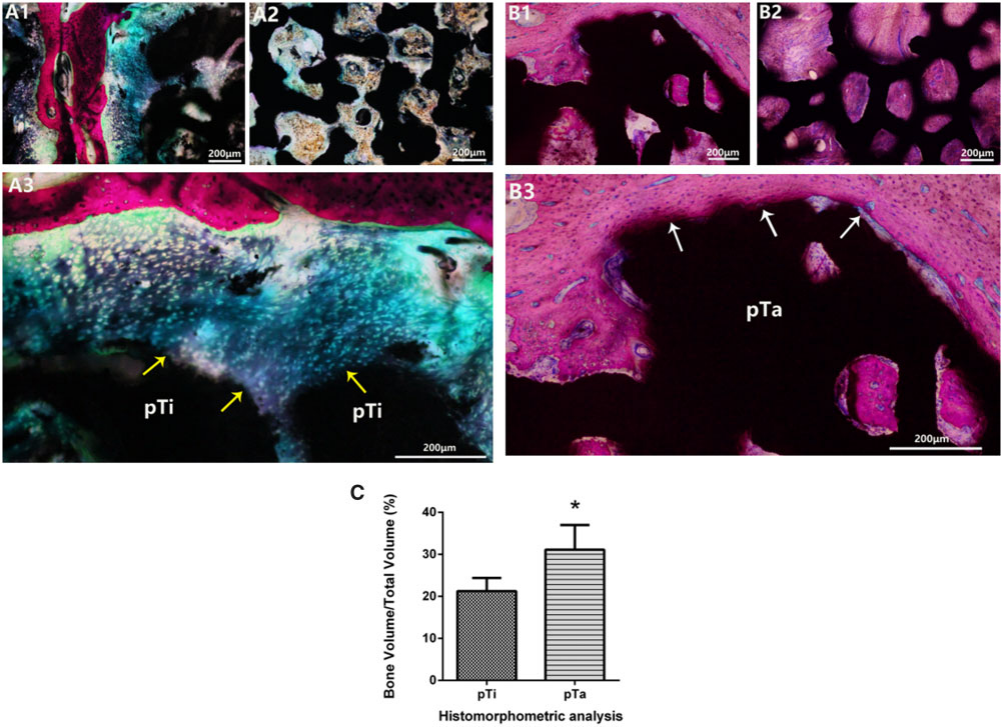
(**A**1–**B**3) Van-Gieson staining of histological sections of the pTi and pTa implants at 12 weeks post-operation (A1–A3 were pTi groups; B1–B3 were pTa groups). the tissue stained with red and purple showed newly formed bone and cartilage, respectively. The tight connection at the interface between the newly formed bone and the implant (marked by white arrows), indicating that the newly formed bone was biologically bound to the implant. (**C**) Histomorphometric analysis of the pTi and pTa implants at 12 weeks post-operation. Asterisks (*) indicate statistical significance compared with the pTi group, *P* < 0.05. Scale bar = 200 μm.

Bone implantation experiments were performed in the hip joint of goats, in order to further evaluate the pTa osseointegration capability through histology. After 12 weeks of implantation, a large amount of newly formed cartilage (marked by purple arrows) could be seen around pTi, but merely few newly formed bones adhered to pTi (Fig. 6A1). Meanwhile, the porous pores were filled with fibrous tissue or few new-born chondrocytes, but no new bone formation was observed (Fig. 6A2). There were a large number of newly formed bones around pTa and in porous pores, which was closely attached to the pTa (Fig. 6B1 and B2). The Van-Gieson staining results that the pTa group had better osseointegration than the pTi group. The pTa directly contacted with the newly formed bone tissue (marked by white arrows), which has good osseointegration. The histomorphometric analysis revealed that the volumes of newly formed bone were significantly higher in the pTa group compared with the pTi group at 12 weeks (Fig. 6C). The new bone tissue has grown to the pTa interior to enhance the connection with the bone tissue, promotes the regeneration and reconstruction of bone tissue, and effectively enhancing the strength and stability of the implant. The above results revealed that the Ta coating substantially improved the osteogenesis and osseointegration property of porous SiC scaffolds, which is consistent with the results of *in vitro* experiments.

These results show that porous SiC scaffolds coated with bioactive Ta metal have good 3D connected structure, excellent biocompatibility and mechanical properties, and are very promising bone replacement implant products.

## Conclusion

The porous SiC scaffolds are successfully coated with a uniform and adherent bioactive Ta film using the CVD method. The prepared pTa has an excellent 3D pore structure and high porosity, and the Ta coating has high-purity and a high bonding strength. Mechanical and histology experiments show that the novel pTa metal has excellent mechanical and osseointegration properties, which indicates its potential for orthopaedic applications. Thus, an alternative material for medical implants for bone substitutions based on high-strength and low density biomorphic porous SiC ceramics coated with bioactive Ta metal is demonstrated herein, and this material combines the characteristics of both materials into a new product with enhanced mechanical and biochemical properties.

## Funding

This work was supported by the National major research and invention programme of the thirteenth of China (no. 2016YFC1102000), and the Dalian Science and Technology Innovation Fund Project (no. 2018J11CY030).

*Conflict of interest statement.* None declared.
